# Evaluation of Xpert MTB/RIF Ultra performance on bronchoalveolar lavage fluid samples for pulmonary tuberculosis diagnosis

**DOI:** 10.36416/1806-3756/e20260059

**Published:** 2026-08-13

**Authors:** Maria Cristina Valim Vieira, André Kulzer Santos, Raimunda Sinthia Lima de Braga, Marina Scheffer de Souza, Gean Souza Ramos, Allanamara Pereira Marinho, Denise Rossato Silva

**Affiliations:** 1. Programa de Pós-Graduação em Ciências Pneumológicas, Universidade Federal do Rio Grande do Sul - UFRGS - Porto Alegre (RS) Brasil.; 2. Faculdade de Medicina, Universidade Federal do Rio Grande do Sul - UFRGS - Porto Alegre (RS) Brasil.; 3. Serviço de Pneumologia, Hospital de Clínicas de Porto Alegre - HCPA - Porto Alegre (RS) Brasil.

**Keywords:** Tuberculosis, pulmonary/diagnosis, Bronchoalveolar lavage fluid, Tuberculosis, pulmonary/microbiology

## Abstract

**Objective:**

In cases in which tuberculosis is not diagnosed microbiologically through sputum samples, bronchoscopy has been the conventional method to increase diagnostic rates. Few studies have evaluated the performance of the Xpert MTB/RIF Ultra assay on BAL fluid samples. We therefore sought to evaluate the performance of Xpert MTB/RIF Ultra on BAL fluid samples for pulmonary tuberculosis diagnosis in a Brazilian city with a high incidence of tuberculosis.

**Methods:**

This was a cross-sectional study performed at a tertiary care university hospital in southern Brazil. Patients > 18 years of age with respiratory symptoms suggestive of pulmonary tuberculosis were invited to participate. Test results were collected, including smear microscopy, mycobacterial culture, and Xpert MTB/RIF Ultra on BAL fluid samples.

**Results:**

During the study period, 161 patients met the inclusion criteria and were therefore included in the analysis. With culture being considered the gold standard, the sensitivity, specificity, positive predictive value, and negative predictive value of Xpert MTB/RIF Ultra on BAL fluid samples were 97.3% (95% CI, 90.6-99.7), 94.3% (95% CI, 87.1-98.1), 93.5% (95% CI, 86.0-97.1), and 97.6% (95% CI, 91.3-99.4), respectively.

**Conclusions:**

The Xpert MTB/RIF Ultra assay showed high sensitivity and specificity on BAL fluid samples from individuals with presumptive pulmonary tuberculosis. This demonstrates that the test is a useful tool for pulmonary tuberculosis diagnosis under programmatic conditions.

## INTRODUCTION

Tuberculosis diagnosis is challenging in patients who are unable to produce sputum. In a systematic review including data from 114 studies,^1^ approximately one quarter of patients with presumed tuberculosis were reported to be unable to produce sputum for diagnosis. In such cases, there is an increased risk of delayed diagnosis and treatment.^2^

In cases in which tuberculosis is not diagnosed microbiologically through sputum samples, bronchoscopy has been the conventional method to increase diagnostic rates. The use of the Xpert MTB/RIF Ultra assay on BAL fluid samples has emerged to increase the diagnostic yield (in comparison with smear microscopy and culture). However, only a few studies have evaluated the performance of Xpert MTB/RIF Ultra on BAL fluid samples.^3^ ^-^ ^5^ Two studies,^3^ ^,^ ^4^ both of which were conducted in China, demonstrated that the Xpert MTB/RIF Ultra assay on BAL fluid samples improved diagnostic accuracy. Studies have shown that the Xpert MTB/RIF Ultra assay on BAL fluid has high sensitivity and specificity for detecting *Mycobacterium tuberculosis*. In a cross-sectional, retrospective diagnostic accuracy study conducted in Colombia,^5^ Xpert MTB/RIF showed a sensitivity of 91.67% and a specificity of 90.09%. No studies in Brazil have evaluated the diagnostic performance of the Xpert MTB/RIF Ultra assay on BAL fluid samples. Therefore, the objective of the present study was to evaluate the performance of Xpert MTB/RIF Ultra on BAL fluid samples for pulmonary tuberculosis diagnosis in a Brazilian city with a high incidence of tuberculosis. 

## METHODS

### Study design and location

We conducted a cross-sectional study at a tertiary care university hospital (the *Hospital de Clínicas de Porto Alegre*, located in the city of Porto Alegre, in southern Brazil). The city of Porto Alegre has a tuberculosis incidence of 64.8 cases/100,000 population.^6^ The study was approved by the Research Ethics Committee of the *Hospital de Clínicas de Porto Alegre* on October 10, 2024 (Protocol no. 20240329). 

### Patients and data collection

Patients > 18 years of age presenting with respiratory symptoms suggestive of pulmonary tuberculosis-such as cough for > 2 weeks and cough of any duration accompanied by constitutional symptoms (such as fever for at least 3 days, night sweats, and weight loss of at least 3 kg in the previous month), as well as hemoptysis-and undergoing bronchoscopy with BAL fluid collection were invited to participate in the study. Patients with a history of tuberculosis were excluded. Pulmonary tuberculosis was diagnosed in accordance with the 2021 Brazilian Thoracic Association consensus statement.^7^

A standardized form was completed for each patient, including the following: demographic data (including sex, age, and race); symptoms (including cough, fever, night sweats, hemoptysis, weight loss, dyspnea, and chest pain); smoking history; alcohol use; drug use; and comorbidities. Test results were also collected, including smear microscopy, mycobacterial culture, and Xpert MTB/RIF Ultra on BAL fluid samples, as well as chest X-ray findings (categorized as cavitary disease, infiltrate, consolidation, fibrotic changes, or a miliary pattern). 

### Statistical analysis

Data analysis was performed with the Predictive Analytics Software package, version 18.0 (SPSS Inc., Chicago, IL, USA) and the MedCalc statistical software package, version 16.4.3 (MedCalc Software Ltd., Ostend, Belgium). Data were presented as number of cases and proportions, mean ± standard deviation, or median [interquartile range]. Categorical comparisons were performed with the chi-square test with Yates’ correction (when appropriate) or with Fisher’s exact test. Continuous variables were compared by means of the t-test or the Wilcoxon test. A two-sided value of p < 0.05 was considered significant for all analyses. 

For the diagnosis of pulmonary tuberculosis, positive mycobacterial culture results were considered the gold standard. Trace results were classified as positive. On the basis of culture results, we calculated the sensitivity, specificity, positive predictive value, and negative predictive value (with 95% CIs) of the Xpert MTB/RIF Ultra assay on BAL fluid samples for the diagnosis of tuberculosis. We constructed ROC curves for the Xpert MTB/RIF Ultra assay on BAL fluid samples. 

In order to calculate the sample size, we considered that the mean sensitivity of Xpert MTB/RIF Ultra was 85%.(8) Therefore, with a 95% confidence interval and a power of 80%, the required sample size was calculated at 100. 

## RESULTS

During the study period, 161 patients met the inclusion criteria and were therefore included in the analysis. The characteristics of the study population are shown in Table 1. The mean age was 55.5 ± 17.1 years. Of the 161 patients included in the present study, 61.5% were male and 78.3% were White. The most common symptoms were cough, in 59 patients (36.6%); dyspnea, in 49 (30.4%); fever, in 38 (23.6%); and weight loss, in 32 (19.9%). Twenty-one patients (13.0%) were living with HIV, and 29 (18.0%) had diabetes mellitus. The most common chest X-ray findings were infiltrates, in 88 patients (54.7%); consolidation, in 68 (42.2%); fibrotic changes, in 26 (16.1%); and cavitation, in 12 (7.5%). 

**Table 1:** Characteristics of the study population.^a^

Characteristic	N = 161
Demographics	
Age, years	55.5 ± 17.1
Male	99 (61.5)
White	126 (78.3)
Symptoms	
Cough	59 (36.6)
Weight loss	32 (19.9)
Dyspnea	49 (30.4)
Fever	38 (23.6)
Night sweats	16 (9.9)
Hemoptysis	10 (6.2)
Current smoking	37 (23.0)
Alcohol abuse	33 (20.5)
Drug use	11 (6.8)
HIV infection	21 (13.0)
Diabetes mellitus	29 (18.0)
Radiographic patterns	
Cavitation	12 (7.5)
Consolidation	68 (42.2)
Infiltrates	88 (54.7)
Fibrotic changes	26 (16.1)
Miliary pattern	2 (1.2)
AFB-positive BAL fluid	45 (28.1)
Culture-positive BAL fluid	50 (31.0)
Xpert MTB/RIF Ultra-positive BAL fluid	77 (47.8)
Trace	13/77 (16.9)
Rifampin resistance	3 (1.9)

a Data presented as mean ± SD; and n/N (%), i.e., number of cases with that characteristic/total number of cases (proportion),

Among the 161 patients suspected of having pulmonary tuberculosis, there were 87 who were culture negative and 74 who were culture positive. Of the 87 culture-negative patients, 5 had positive Xpert MTB/RIF Ultra tests, which had trace results. The patients were therefore treated for tuberculosis, the clinical and radiological picture having also been taken into consideration. Of the 74 culture-positive patients, 72 had positive Xpert MTB/RIF Ultra tests; the two false-negative patients were treated for tuberculosis because the clinical and radiological picture was strongly suggestive of tuberculosis. 

**Figure 1:**
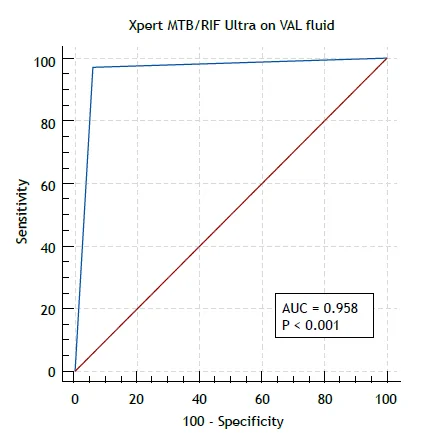
ROC curve for detection of tuberculosis by means of the Xpert MTB/RIF Ultra assay on BAL fluid samples. The area under the ROC curve (AUC) was = 0.958 (95% CI, 0.91-0.98; p < 0.001).

With culture being considered the gold standard, the sensitivity, specificity, positive predictive value, and negative predictive value of the Xpert MTB/RIF Ultra assay on BAL fluid samples were 97.3% (95% CI, 90.6-99.7), 94.3% (95% CI, 87.1-98.1), 93.5% (95% CI, 86.0-97.1), and 97.6% (95% CI, 91.3-99.4), respectively. The area under the ROC curve was = 0.958 for the Xpert MTB/RIF Ultra assay on BAL fluid samples (95% CI, 0.91-0.98; p < 0.001; Figure 1). 

## DISCUSSION

In the present study, we evaluated the performance of the Xpert MTB/RIF Ultra assay on BAL fluid samples. We demonstrated that Xpert MTB/RIF Ultra has high sensitivity (97.3%), specificity (94.3%), positive predictive value (93.5%), and negative predictive value (97.6%) on BAL fluid samples from patients with suspected pulmonary tuberculosis. 

Smear-negative pulmonary tuberculosis poses a diagnostic challenge, depending on a high index of suspicion. Overall, 40-60% of suspected cases of pulmonary tuberculosis will have acid-fast bacilli smear-negative results.^9^ This diagnostic difficulty can lead to delayed diagnosis, a worse prognosis, and increased mortality, especially in populations with a high prevalence of HIV infection.^10^ ^,^ ^11^ In addition, despite a lower bacterial load, smear-negative pulmonary tuberculosis cases are associated with 10-20% of tuberculosis transmission at the population level.^10^ ^,^ ^11^

We found high sensitivity and specificity for the Xpert MTB/RIF Ultra assay on BAL fluid samples, a finding that is similar to those reported in previous studies.^4^ ^,^ ^5^ In a prospective study conducted in China,^4^ the authors evaluated the performance of Xpert MTB/RIF Ultra for detection of *M*. *tuberculosis* on BAL fluid samples from people living with HIV; they found a sensitivity of 96% and a specificity of 92%. In a cross-sectional retrospective study conducted in Colombia and evaluating the use of Xpert MTB/RIF on BAL fluid samples for the diagnosis of pulmonary tuberculosis in sputum-scarce or smear-negative patients,^5^ a total of 283 adults were included, and Xpert MTB/RIF showed a sensitivity of 91.67%, a specificity of 90.09%, a positive predictive value of 67.69%, and a negative predictive value of 97.95%. The study was the first to evaluate the accuracy of Xpert MTB/RIF in a high-burden South American country. 

One of the limitations of the present study is that we recruited patients from a single center. Furthermore, we had a small number of people living with HIV, and this prevented us from doing subgroup analyses. Despite these concerns, this is the first study in Brazil to evaluate the accuracy of Xpert MTB/RIF Ultra on BAL fluid samples. 

In conclusion, the Xpert MTB/RIF Ultra assay showed a high sensitivity and specificity on BAL fluid samples from individuals with presumptive pulmonary tuberculosis. This demonstrates that the test is a useful tool for pulmonary tuberculosis diagnosis under programmatic conditions. 
